# Prolonged acute mechanical ventilation and hospital bed utilization in 2020 in the United States: implications for budgets, plant and personnel planning

**DOI:** 10.1186/1472-6963-8-242

**Published:** 2008-11-25

**Authors:** Marya D Zilberberg, Andrew F Shorr

**Affiliations:** 1 School of Public Health and Health Sciences, University of Massachusetts, Amherst, MA, USA; 2 Evi *Med *Research Group, LLC, Goshen, MA, USA; 3 Division of Pulmonary and Critical Care, Washington Hospital Center, Washington, DC, USA

## Abstract

**Background:**

Adult patients on prolonged acute mechanical ventilation (PAMV) comprise 1/3 of all adult MV patients, consume 2/3 of hospital resources allocated to MV population, and are nearly twice as likely to require a discharge to a skilled nursing facility (SNF). Their numbers are projected to double by year 2020. To aid in planning for this growth, we projected their annualized days and costs of hospital use and SNF discharges in year 2020 in the US.

**Methods:**

We constructed a model estimating the relevant components of hospital utilization. We computed the total days and costs for each component; we also applied the risk for SNF discharge to the total 2020 PAMV population. The underlying assumption was that process of care does not change over the time horizon. We performed Monte Carlo simulations to establish 95% confidence intervals (CI) for the point estimates.

**Results:**

Given 2020 projected PAMV volume of 605,898 cases, they will require 3.6 (95% CI 2.7–4.8) million MV, 5.5 (95% CI 4.3–7.0) million ICU and 10.3 (95% CI 8.1–13.0) million hospital days, representing an absolute increase of 2.1 million MV, 3.2 million ICU and 6.5 million hospital days over year 2000, at a total inflation-adjusted cost of over $64 billion. Expected discharges to SNF are 218,123 (95% CI 177,268–266,739), compared to 90,928 in 2000.

**Conclusion:**

Our model suggest that the projected growth in the US in PAMV population by 2020 will result in annualized increases of more than 2, 3, and 6 million MV, ICU and hospital days, respectively, over year 2000. Such growth requires careful planning efforts and attention to efficiency of healthcare delivery.

## Background

Healthcare institutions today are frequently faced with stretching limited resources. In the US, factors contributing to this are significant and worsening personnel shortages [1-10], reductions in reimbursement [11], and in numbers of hospital and intensive care unit (ICU) beds [12] in the face of growing demand for healthcare [13-15]. After a decade of declines in hospital admissions, since the mid-1990s there has been an annualized 1.5% growth in hospital volume [16]. Similarly, total inpatient days have stabilized over the last 10 years at about 197 million [16]. These events have occurred against the backdrop of hospital and bed closures, resulting in a drop in hospital beds between 1995 and 2005 from 3.3 to 2.7 per 1,000 population [17]. Currently, nearly half of Emergency Departments (EDs) are reporting operating at or over capacity, and one-third report some time on diversion, the most frequent reason being the lack of staffed critical care beds [16]. This is not surprising, since the number of ICU beds has remained essentially unchanged between 1991 and 2004 [18].

We recently described a novel sub-population of patients on mechanical ventilation (MV), those requiring prolonged acute mechanical ventilation (PAMV, defined as MV for ≥ 96 hours), who, though comprising 1/3 of all patients on MV, utilize 2/3 of the associated hospital resources. [19]. Numbering about 300,000 cases in 2003, and accounting for nearly 7 million hospital days and $16 billion in hospital costs annually [19], the PAMV volume is projected to more than double by year 2020 [20]. This growth, on the par with that among patients with sepsis and severe sepsis [21-23], is far greater than previously predicted and well outpaces the increase seen in hospital discharges overall [3,4,16]. In the absence of information projecting healthcare utilization for such resource-intensive groups of patients, the limited hospital resources cannot be expected to keep pace.

In the current study, we have quantified the expected US-specific hospital bed resources and associated costs of providing care to the adult PAMV patients in year 2020. Specifically, we have calculated bed day numbers and costs within the relevant care strata, including MV, ICU, and hospital days that these patients may occupy. Additionally, since PAMV patients are roughly 50% more likely to be discharged to a skilled nursing facility (SNF) than those requiring shorter term MV [19], we have projected the numbers that will likely require such subacute care following their acute hospitalization.

## Methods

No human subjects were enrolled in the study, and, thus, the study was exempt from regulations guiding protection of human subjects. We developed a model utilizing publicly available inputs, and tested the robustness of the outcome estimates in multivariate analyses. All calculations were performed in Microsoft Excel (Microsoft Corporation, Redmond, WA) and Crystal Ball^® ^software (Decisioneering, Inc., Denver, CO).

### Model overview and structure

We utilized the projected number of adult PAMV cases in year 2020 [20] to allocate total bed days and costs in the following annualized categories: MV days, non-MV ICU days, total ICU days, non-ICU hospital days and total hospital days. Additionally, based on previously reported data [19], we projected the anticipated annualized volume of discharges to a SNF under an assumption that hospital care delivery remains constant through year 2020.

### Model inputs

Model input parameters and their sources are depicted in Table 1.

**Table 1:** Model Parameter Estimates and Sources

**Input variable**	**Point estimate**	**Range (95% CI)**	**Source/Calculation**
**VOLUME AND BED DAYS**			

Annual adult PAMV volume (number of discharges)			

2000	252,577	N/A	[25]

2020	605,898	456,695 to 779,806	[20]

Incremental LOS by level of service (days)			

MV	6	4 to 11	[29]

Non-MV ICU	3	2 to 4	Total ICU – MV [29]

Total ICU	9	6 to 15	[29]

Non-ICU hospital	8	5 to 11	Median hospital LOS [19] – ICU LOS [29]

Total hospital	17	11 to 26	[19]

			

**COSTS **(2008 $US)			

Cost of MV day	$5,811	$5,050 to $11,374	[31]*

Cost of non-MV ICU day	$2,880	$1,219 to $4,541	[12]^†^

Cost of non-ICU hospital day	$1,170	$827 to $1,513	[12]^†^

PAMV is prolonged acute mechanical ventilation. LOS is length of stay. MV is mechanical ventilation. ICU is intensive care unit. CI is confidence interval. N/A is not applicable.

*Calculated as a weighted average cost based on reference 31 and utilized extreme values for the sensitivity range inflated to year 2008 $US.

^†^Based on reference 12, calculated as the average of the values computed by each reported method to arrive at the point estimates for the model, and varied the inputs across the corresponding ranges; inflated to year 2008 $US.

#### Annual PAMV volume in referent year (2000) and projected to year 2020

The source for this was a recent study based on the numbers from the Nationwide Inpatient Sample (NIS) [24,25] focusing on adult discharges with the ICD-9-CM procedure code 96.72 (MV for 96 hours or longer) [20]. This study calculated both age-adjusted and condition-specific changes in PAMV incidence and, projecting them out to year 2020, reported that the number of PAMV discharges from the US hospitals can be expected to rise from 252,577 in 2000 to 605,898 (95% confidence interval [CI] 456,695 to 779,806) in 2020 [20].

#### Hospital services utilization parameters

While a previous study determined the median hospital length of stay (LOS) in this population to be 17 days (interquartile range [IQR] 25–75 11 to 26), corresponding components of this utilization, i.e., duration of MV or of ICU stay, were not quantified [19]. Since by virtue of spending at least 96 hours on MV the PAMV patients fall outside the estimated ranges for either acutely-ventilated populations [26,27] or those on prolonged MV [28], we developed the following approach to quantifying the MV and ICU components of their hospital utilization:

1). We chose to focus on a population of patients who require the longest time of MV during their acute ICU stay, the groups with adult respiratory distress syndrome (ARDS) [26]. The main rationale for this is that their median time on MV is consistently estimated at > 96 hours [26,29]. Adding validity to this approach are the similarities in both the median age and hospital mortality among PAMV patients (35%) and those reported for the ARDS population [19,29,30]. Finally, ARDS was a frequently coded coexistent condition in the PAMV cohort with the prevalence of 15% [19], though misclassification was possible, given the administrative nature of the data set.

2). Having made this choice, we developed estimates of MV and ICU duration for this group based on a recent robust and generalizable cohort study. The study by Rubenfeld was a large prospective cohort study conducted in King County, Washington, where 1,113 patients with acute lung injury (ALI) and ARDS were enrolled [29]. In this study, the median MV time was 5.3 days (IQR 25–75 2.1–10.8) and median ICU LOS 7.8 days (IQR 25–75 3.7–14.3). From these durations of individual components of hospital stay we were able to derive the following parameter estimates: a). Duration of MV (5.3 days as reported by Rubenfeld [29]), or 37.9% of the overall hospital LOS; b). ICU LOS (7.8 days as reported by Rubenfeld [29]); c). Duration of ICU without MV (total ICU LOS – MV duration), or 17.9% of the overall hospital LOS; and d). Non-ICU hospital LOS (median hospital LOS for the PAMV cohort [19] – ICU LOS reported by Rubenfeld [29]), or 44.3% of the overall hospital LOS.

3). In the final step, based on our previous report quantifying the median hospital LOS in the PAMV population to be 17 days [19], we computed the number of days spent in each stratum of care by applying the proportions of time spent in each of the strata by the ALI patients as reported in the Rubenfeld study [29]. Each of the durations was rounded to the nearest integer.

#### Hospitalization component cost estimates

For the cost of MV we utilized the study by Dasta and colleagues, which quantified average daily costs for a patient on MV through day 14 of MV. In this study using data from 2002 the investigators demonstrated that day 1 of MV is substantially more costly than all subsequent days. To arrive at the point estimate of a daily cost for MV we calculated a weighted average cost for a general ICU patient (found under the rubric of "Total, $" in Table 4 [31]) in the Dasta paper ($3,948) and utilized extreme values ($3,431 to $7,728) around that for the sensitivity range, all inflated to 2008 $US as described below [31].

For both the non-MV ICU day and non-ICU hospital day costs we went to a recent report from Halpern and colleagues, who, based on two distinct sources, calculated a plausible range of these costs in year 2000 [12]. We averaged each range to arrive at the point estimates for the model ($1,696 for non-MV ICU day and $689 for non-ICU hospital day) and varied the inputs across the corresponding ranges ($718 to $2,674 for non-MV ICU day and $487 to $891 for non-ICU hospital day) [12].

Because variable costs are more likely than the fixed to be impacted by population growth, and because variable costs represent only 14% of total hospital costs [32], we performed a sensitivity analysis to arrive at the potential growth in this cost component specifically. Since it remains unclear how much the projected PAMV growth will impact the need to expand hospital plants, equipment and personnel, this analysis represents the absolute minimum of the incremental hospital expenditure that may be expected in conjunction with this growth.

All costs were inflated to year 2008 $US using the hospital and related services component of the Consumer Price Index (CPI) [33]. To inflation-adjust our 2020 cost estimates we calculated the most recent 10-year historic average annual inflation rate within the hospital and related services component of CPI (8.5%) and applied that historic growth to our projected number. We varied this annual inflation rate between 6% and 12% in the simulations. No discounting was required since the expenditures we are quantifying are to take place in the future.

#### Volume of discharges to SNF estimate

This was based on the previous finding that 36% of all PAMV patients are discharged from the hospital to a SNF [19]. This proportion was multiplied by the projected number of PAMV cases in 2020 to arrive at the base case, and the bounds of the 95% CI of this estimate to generate the corresponding 95% CI.

#### Monte Carlo Simulations

Because of uncertainties surrounding some of the parameter estimates in the model, we performed Monte Carlo simulations to test the precision of our estimates. We varied our inputs across their corresponding 95% CIs to generate the 95% CIs around the outcome estimates. Each outcome estimate was tested in 10,000 trials. The probability distributions used for the input parameters were triangular for the annual PAMV volume and log-normal for the LOS estimates in each of the strata; in the case of the latter log-normal was used as the best fit for non-parametric data. For cost inputs, we utilized a log-normal distribution for the cost of MV day, since this was based on the non-parametrically distributed range, where early days are more costly that those later on in the hospitalization [31]. The remaining cost estimates, as well as the annual inflation rate, were varied across the normally distributed ranges. Point estimates were calculated as the mean value of the 10,000 trials and the 95% CIs represent 2.5^th ^and 97.5^th ^percentiles of the distributions.

## Results

**Figure 1:**
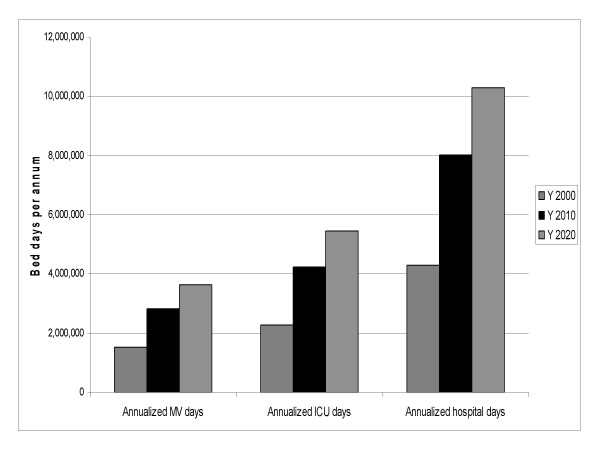
**Projected Annual Hospitalization Days in 10-year Increments Spent by a Patient on Prolonged Acute Mechanical Ventilation (PAMV) in Various Strata of Hospital Care**. ICU is intensive care unit. MV is mechanical ventilation. Y is year.

Over 50% of the duration of hospitalization among critically ill patients with respiratory failure is spent in the ICU (Table 1). In this care stratum, the majority of the time is allocated to MV support (37.9% of the total hospitalization or 68% of the entire ICU time). In the setting of these time allocations, the year 2000 estimate for corresponding bed use is approximately 1.5 million MV days, 750,000 non-MV ICU days, and over 2 million non-ICU hospital days. Given the projected increase of the PAMV population to approximately 490,000 in year 2010 and to over 600,000 cases in year 2020 [20], the corresponding expected absolute increases in the care stratum-specific bed days are an additional 1.3 and 2.1 million MV days, 2.0 and 3.2 million ICU days and 3.7 and 6.0 million total hospital days in 2010 and 2020, respectively (Figure 1). Put another way, we anticipate the bed demands to increase from 1.5 to 3.6 million for MV, from 2.3 to 5.5 million for ICU, and from 4.3 to 10.3 million for annualized hospital days between the years 2000 and 2020 in the population requiring PAMV (Table 2). Additionally, discharges to SNF can be expected to rise from 91,000 in year 2000 to nearly 220,000 by year 2020 (Table 2).

**Table 2:** Outcomes* ^†^

**Outcome**	**Point Estimate**	95% confidence interval*
Annual MV		

2000 days	1,515,462	1,231,585 to 1,829,041

2020 days	3,635,388	2,735,290 to 4,838,746

2020 costs	$42.7	$30.2 to $64.9

Annual non-MV ICU		

2000 days	757,731	618,896 to 913,025

2020 days	1,897,694	1,373,865 to 2,437,155

2020 costs	$10.6	$7.3 to $16.2

Total annualized ICU		

2000 days	2,273,293	1,957,030 to 2,616,069

2020 days	5,453,082	4,246,669 to 7,020,137

2020 costs	$53.2	$39.0 to $78.7

Annual non-ICU hospital		

2000 days	2,020,616	1,658,582 to 2,445,627

2020 days	4,847,184	3,648,522 to 6,444,697

2020 costs	$11.5	$8.0 to $17.3

Total annualized hospital		

2000 days	4,293,809	3,811,826 to 5,323,280

2020 days	10,300,266	8,089,043 to 13,042,347

2020 costs	$64.7	$48.6 to $93.7

Total annualized discharges to SNF		

2000	90,928	N/A

2020	218,123	177,268 to 266,739

*Costs are in billions of $US; 95% confidence intervals estimated utilizing Monte Carlo simulations.

^†^Annual inflation rate of 8.5% calculated by averaging the 10-year historical inflation rate in the hospital and related care component between 1998 and 2008 [33].

MV is mechanical ventilation. ICU is intensive care unit. SNF is skilled nursing facility.

N/A is not applicable.

The results of the Monte Carlo simulations are also depicted in Table 2, showing 95% confidence intervals around the point estimates for each stratum of care. Thus, the total volume of ICU days can be expected to range from 4.2 to 7.0 million days in year 2020, and the total hospital days between 8.1 and 13.0 million.

In terms of costs, PAMV population can be expected to consume a total of $42.7 (95% CI $30.2 to $64.9) billion, $10.6 (95% CI $7.3 to $16.2) billion, and $11.5 (95% CI $8.0 to $17.3) billion in MV, non-MV ICU and non-ICU hospital costs, respectively (Table 2). In other words, the total projected costs of ICU care are $53.2 (95% CI $39.0 to $78.7) billion and the total costs of hospital care for the PAMV population are $64.7 (95% CI $48.6 to $93.7) billion in year 2020. In a sensitivity analysis quantifying variable costs only, the hospital costs consumed by PAMV were $6.0 (95% CI $4.2 to $9.1), $1.5 (95% CI $1.0 to $2.3), and $1.6 (95% CI $1.1 to $2.4) billion in MV, non-MV ICU and non-ICU hospital costs, respectively, yielding the total annual hospital bill of $9.1 (95% CI $6.8 to $13.1) billion.

**Figure 2:**
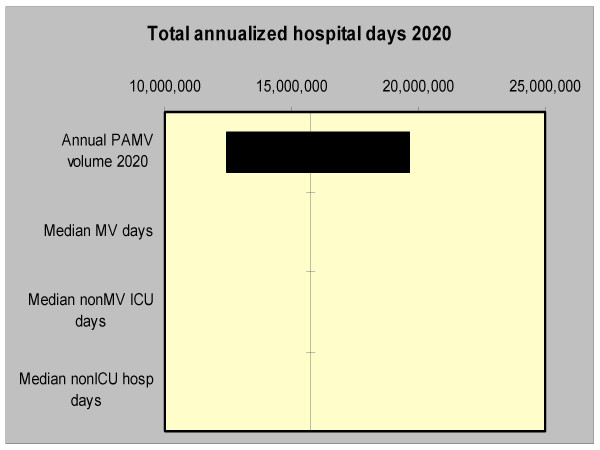
**Tornado diagram. The solid vertical line represents the point estimate for the projected annualized hospital days occupied by PAMV patients in year 2020. The horizontal bars represent the range of this difference when the corresponding single input is varied across its designated range with all other input parameters held constant**. PAMV is prolonged acute mechanical ventilation. MV is mechanical ventilation. ICU is intensive care unit.

**Figure 3:**
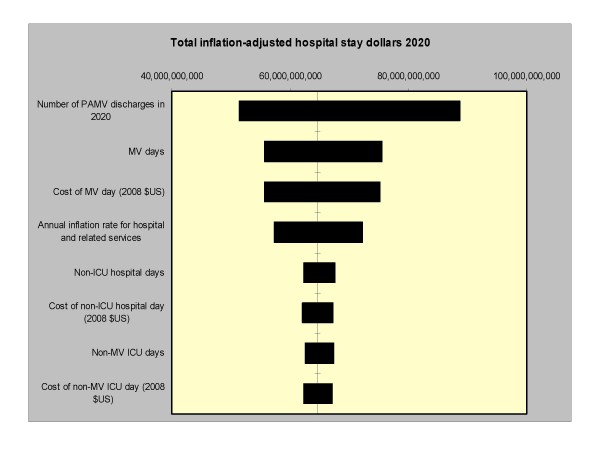
**Tornado diagram. The solid vertical line represents the point estimate for the projected annualized hospital costs for PAMV patients in year 2020. The horizontal bars represent the range of this difference when the corresponding single input is varied across its designated range with all other input parameters held constant**. PAMV is prolonged acute mechanical ventilation. MV is mechanical ventilation. ICU is intensive care unit.

Importantly, an analysis of factors contributing to the uncertainty in the model estimates quantifying bed occupancy revealed that the imprecision of the projected number of PAMV cases in year 2020 was responsible for nearly all of the uncertainty, while the estimates of the LOS did not make a significant contribution in the model quantifying bed usage (Figure 2). In the model quantifying costs, on the other hand, while the imprecision of the projected number of PAMV cases in year 2020 was responsible for the majority of the outcome estimate uncertainty, other factors also contributed substantial proportions of the imprecision (Figure 3).

## Discussion

We have shown that, based on previous projections for their growth over time in the US, the PAMV population can be expected to more than double their acute hospital bed use in each of the care strata, as well as the use of SNF as a discharge destination, resulting in 3.6 million MV, 5.5 million ICU and 10.3 million total hospital days in year 2020. While in the most modest growth scenario, this change will be just under 2-fold, under the scenario of greatest anticipated growth, the 2020 numbers may be as high as 4.8 million, 7.0 million, and 13.0 million bed days for MV, ICU and hospital, respectively. Similarly, the corresponding inflation-adjusted costs can be expected to be over $42, $53, and $64 billion for MV, ICU and total hospital costs, respectively. Additionally, we project that, in the absence of changes in care flow of the PAMV patients, the volume of SNF discharges in this population can be expected to be between roughly 177,000 and 267,000.

The landscape of hospital-based care in the US has undergone profound changes over the last 25 years. Following a period of exuberant growth peaking in the 1970s at ~7,200 hospitals [34], the subsequent decades have seen a steep reduction in inpatient utilization, and a decrease in the number of hospitals to just under 5,500 [12]. This decline has continued in the face of attempts to rein in unprecedented healthcare expenditures, where hospital care accounts for ~1/3 of the spending [14]. Indeed, in 2005 there were just over 800,000 beds in community hospitals across the US, or 2.71 beds per 1,000 population, the lowest it has been in over two decades [16]. This decrease is paradoxic, given that in-patient admissions have been steadily on the rise since the mid-1990s, and the average hospital LOS has not decreased appreciably since year 2000 [16,25]. In contrast, the number of ICU beds has increased by 26.2% from 69,300 to 87,400 between 1985 and 2000 [12], an approximate annual rate of growth of 1.7%. If constant through year 2020, this will result in a cumulative growth of 34% from the 2000 bed number. It is unlikely that this modest rise in the number of beds will be able to accommodate a nearly 140% growth in bed demand by the PAMV population, since already today among the hospitals reporting ED diversion, the number one reason for this is the lack of staffed ICU beds [16]. Commensurate with this growth in bed utilization, as well as in view of expected inflation rates, the annual spending on hospital care alone in this population may be expected to rise from $16 billion in 2003 to over $64 billion by 2020.

How can we address this projected rise in demand in ICU and hospital services attributed to the growth in PAMV population? In addition to making appropriate allocation, construction and staffing decisions, we need to focus on optimizing the efficiency of care delivered to this and other growing populations [35,36]. Starting upstream, perhaps one lesson is to increase our attention to prevention of complications that render one susceptible to a prolonged critical illness. Our model suggests that this would be most impactful, given that it is the number of PAMV patients, and not the utilization at the individual level, that is the strongest driver of the cumulative bed occupancy. Short of this, and once critical illness has occurred, adoption of such practices as lung-protective ventilation [37], ventilator-associated pneumonia prevention [38], sterile technique for central catheter insertion [39], and limiting exposure to such potentially complicating agents as allogeneic blood components [40-43] needs to be locally optimized. Alternatively, in appropriate cases, a stronger emphasis may be placed on end-of-life discussions, resulting in elimination of care deemed unnecessary. Regionalization of care is worth considering. However, while data suggest improved volume-outcomes relationship in hospital survival for patients on MV, no such link has been established for utilization outcomes [27]. Finally, since a substantial body of information provides evidence that early tracheostomy may increase patient comfort, has limited potential for harm and is at the same time associated with a reduced time on MV and in the ICU [44], it may be a viable strategy in at-risk patients. However, should an early tracheostomy become the standard of care, there will almost certainly be an increased need in SNF facilities. In general, in view of major improvements in outcomes among very low birth weight infants, and the attendant increase in hospital resource utilization, some lessons in how to manage PAMV population growth may be derived from the experiences of neonatal ICUs across the US [45].

Our study has multiple limitations. Although the procedure code 96.72 has been used previously to identify the PAMV population [19,20], neither its validity nor accuracy has been formally evaluated. The numbers we cite are based on model simulations, and as such are sensitive to the accuracy of input parameters. To incorporate the uncertainty of input values into our estimates, we performed Monte Carlo simulations and sensitivity analyses, providing 95% confidence bounds for all of the estimates. The estimates for the LOS in every stratum of hospital care are derived from a single study of patients with acute lung injury, and thus may not be generalizable to all PAMV patients. However, given the fact that 15% of all PAMV patients have a diagnosis code for ARDS, that hospital mortality in PAMV patients is similar to that seen in ARDS, and that the hospital LOS associated with PAMV has been found to be similar to that seen in ARDS [19,29], our estimates are likely to be in the right range. Additionally, because the studies from which these critical inputs were derived reported the LOS components as median rather than mean values, our estimates likely underestimate the true LOS. Another factor that adds to the internal validity of this assumption is that when the hospital costs reported for PAMV population in 2003 [19] are aggregated and inflated to 2020 $US to reflect the projected population, the total hospital costs ($68.1 billion) are quite similar to those seen in the model ($64.7 billion). Additionally, we have found that the bed utilization model is most sensitive to the accuracy of the predicted PAMV numbers, and not to the LOS inputs. A limitation of using total hospital costs is worth discussing as well. Since it has been shown that only a small fraction of the total hospital costs is due to the variable component of costs [32], our numbers likely overestimate the actual incremental increases in expenditures necessary to care for the growing PAMV population in the future. However, even if a conservative estimate of 14% of the total costs is used [32], the total annualized hospital costs associated with the care of the PAMV population will approximate $10 billion in variable hospital costs by 2020. This is a three-fold growth compared to the 2003 estimate for the indirect hospital costs for the care of this population [19]. Because one of the outcomes examined was the volume of SNF discharges, the vague nature of the SNF definition has to be brought up. Since the NIS does not explicitly provide the definition, we assumed that, in accordance with Medicare, a SNF is "a nursing facility with the staff and equipment to give skilled nursing care and/or skilled rehabilitation services and other related health services", and SNF care is defined as "a level of care that requires the daily involvement of skilled nursing or rehabilitation staff and that, as a practical matter, can't be provided on an outpatient basis" [46]. Thus, it is likely that our SNF designation includes long-term acute care facilities, though we cannot definitively state so. Finally, one of the major underlying assumptions of the study is that the patterns of care will remain the same. This assumption is not unreasonable, given that translating evidence into practice can take decades [47]. Thus, monitoring the volume of PAMV patients longitudinally and measures to improve efficiency in their care delivery will assure the accuracy of predictions in the future.

At the same time, our study has a number of strengths. To the best of our knowledge, it is the first study to project hospital utilization and costs by a large and growing resource-intensive population. Furthermore, we have divided this utilization estimate into the various strata of hospital care relevant to making decisions with regard to budgeting, construction, equipment and hospital personnel allocations. For example, given that the number of MV days may double if no measures, such as early tracheostomy or regionalization of care, prove effective at reducing this duration, a hospital may need to include such capital investments as the purchase of additional ventilators in its planning efforts.

In summary, we have demonstrated that hospital bed utilization between years 2000 and 2020 will go from 1.5 to 3.6 million for MV, from 2.3 to 5.5 million ICU, and from 4.3 to 10.3 million annualized hospital days for the population requiring PAMV, and the expected annual hospital costs may be over $64 billion. Our projections put into perspective the fact that efficiency improvements can no longer be viewed as an option, but are a clinical and policy imperative.

## Abbreviations

ICU: intensive care unit; ED: emergency department; PAMV: prolonged acute mechanical ventilation; MV: mechanical ventilation; SNF: skilled nursing facility; CI: confidence interval; LOS: length of stay; IQR: interquartile range; ARDS: adult respiratory distress syndrome; ALI: acute lung injury

## Competing interests

MDZ has no conflict of interest to declare. AFS has no conflict of interest to declare. No funding was received by the authors in conjunction with this work.

## Authors' contributions

MDZ conceived and designed the study, carried out the analyses and interpretation of the results, and participated in drafting of the manuscript. AFS participated in the study design, interpretation of the results and the drafting of the manuscript. Both authors have given final approval of the manuscript.

## Pre-publication history

The pre-publication history for this paper can be accessed here:


